# Transmission of Antimicrobial-Resistant *Staphylococcus aureus* Clonal Complex 9 between Pigs and Humans, United States

**DOI:** 10.3201/eid2703.191775

**Published:** 2021-03

**Authors:** Pranay R. Randad, Jesper Larsen, Hülya Kaya, Nora Pisanic, Carly Ordak, Lance B. Price, Maliha Aziz, Maya L. Nadimpalli, Sarah Rhodes, Jill R. Stewart, Dave C. Love, David Mohr, Meghan F. Davis, Lloyd S. Miller, Devon Hall, Karen C. Carroll, Trish M. Perl, Christopher D. Heaney

**Affiliations:** Johns Hopkins University, Baltimore, Maryland, USA (P.R. Randad, N. Pisanic, C. Ordak, D.C. Love, D. Mohr, M.F. Davis, L.S. Miller, K.C. Carroll, T.M. Perl, C.D. Heaney);; Statens Serum Institut, Copenhagen, Denmark (J. Larsen, H. Kaya);; George Washington University, Washington, DC, USA (L.B. Price, M. Aziz);; Tufts University, Boston, Massachusetts, USA (M.L. Nadimpalli);; University of North Carolina at Chapel Hill, Chapel Hill, North Carolina, USA (S. Rhodes, J.R. Stewart);; Rural Empowerment Association for Community Help (REACH), Warsaw, North Carolina, USA (D. Hall);; University of Texas Southwestern Medical Center, Dallas, Texas, USA (T.M. Perl)

**Keywords:** *Staphylococcus aureus*, livestock-associated diseases, antimicrobial resistance, industrial hog operations, pigs, zoonotic transmission, infectious disease transmission, zoonoses, AMR, multidrug-resistant bacteria, food safety, bacteria, MRSA and other staphylococci, North Carolina, United States

## Abstract

Transmission of livestock-associated *Staphylococcus aureus* clonal complex 9 (LA-SA CC9) between pigs raised on industrial hog operations (IHOs) and humans in the United States is poorly understood. We analyzed whole-genome sequences from 32 international *S. aureus* CC9 isolates and 49 LA-SA CC9 isolates from IHO pigs and humans who work on or live near IHOs in 10 pig-producing counties in North Carolina, USA. Bioinformatic analysis of sequence data from the 81 isolates demonstrated 3 major LA-SA CC9 clades. North Carolina isolates all fell within a single clade (C3). High-resolution phylogenetic analysis of C3 revealed 2 subclades of intermingled IHO pig and human isolates differing by 0–34 single-nucleotide polymorphisms. Our findings suggest that LA-SA CC9 from pigs and humans share a common source and provide evidence of transmission of antimicrobial-resistant LA-SA CC9 between IHO pigs and humans who work on or live near IHOs in North Carolina.

Livestock-associated *Staphylococcus aureus* (LA-SA) has emerged among pigs raised in industrial hog operations (IHOs) and persons who work on or live near IHOs globally, including in the United States (*1*–*4*). IHO workers who are occupationally exposed to pigs are at increased risk for intranasal carriage of *S. aureus*, including methicillin-resistant *S. aureus* (MRSA), multidrug-resistant *S. aureus* (MDRSA), and LA-SA (*3*,*5*). Furthermore, persons exposed to LA-SA are at risk of developing mild-to-severe infections, including skin and soft tissue infections (SSTIs), pneumonia, endocarditis, osteomyelitis, and bacteremia (*5*–*8*). Recent evidence supports emergence of diverse clones associated with IHOs. *S. aureus* clonal complex 9 (CC9), for example, has been reported as a dominant LA-SA lineage in Asia and has been described as an emerging clone in some areas with intensive industrial livestock production in the United States (*9*–*11*).

The population structure and transmission dynamics of emerging LA-SA CC9 strains in the United States remains poorly understood. Previous epidemiologic studies in the top 10 pig-producing counties in North Carolina, the second leading US pig-producing state, showed a high prevalence of LA-SA CC9 nasal carriage among IHO pigs and IHO workers (*3*,*12*). Epidemiologic findings provide support for potential transmission of LA-SA CC9 between IHO workers and their household contacts, including minor children (<18 years of age; IHO minors), based on nasal carriage of LA-SA CC9 with concordant *spa* types at the same time point (*3*). Epidemiologic studies have also identified instances of LA-SA CC9 nasal carriage among community residents with no known exposure to livestock in high-density IHO areas of North Carolina (*2*). Whole-genome sequencing (WGS) analysis provides an opportunity to characterize the population structure and transmission dynamics of LA-SA CC9 in the United States. The objectives of this study were to use WGS and phylogenetic analyses to elucidate the population structure of *S. aureus* CC9 from various regions in North America, South America, Europe, and Asia and to investigate potential transmission of antimicrobial-resistant LA-SA CC9 among IHO pigs and humans who work on or live near IHOs in North Carolina.

## Methods

### Sources of *S. aureus* Isolates from Humans and from Pigs Raised on IHOs in North Carolina

*S. aureus* isolates from IHO pigs were collected from a convenience sample of a single IHO in North Carolina (IHO-1), as described previously (*12*). We collected additional pig samples by hanging a length of undyed, unbleached cotton rope in pig pens of 20 IHOs in North Carolina (IHO-2–IHO-21) (Appendix). Pig isolates were recovered from IHO-2, IHO-3, IHO-4, IHO-5, and IHO-6 for a total of 6 IHOs (IHO-1–IHO-6). Isolates from IHO-2–IHO-6 have not been published previously. The *spa* type for all IHO pig isolates was characterized, as previously described (*12*), and used to assign each isolate to a putative multilocus sequence type (MLST). 

*S. aureus* isolates from humans were collected from participants who were previously enrolled into 1 of 3 separate epidemiologic studies (study 1, study 2, and study 3) and screened for nasal carriage of *S. aureus* (Appendix). Sample collection, sample processing, and *S. aureus* isolation methods were described previously (*1*–*3*). MLST was previously determined for all study 1 isolates (*1*). The *spa* type was previously characterized for study 2 and study 3 isolates and used to assign a putative MLST based on previously published associations between *spa* types and MLSTs (*2*,*3*).

### Selection of *S. aureus* CC9 Isolates for WGS Analysis

A total of 236 putative or MLST-confirmed *S. aureus* CC9 isolates were recovered from IHO pigs (n = 91) and humans (n = 145) in North Carolina during 2011–2016 (Appendix). For this study, a convenience sample of 49 isolates from North Carolina were subjected to WGS analysis, including 10 isolates from pigs raised on 4 different IHOs, 34 isolates from 25 IHO workers, 1 isolate each from 3 IHO minors, and 1 isolate each from 2 community resident adults (Appendix). For comparative purposes, we also included an international collection of 32 *S. aureus* CC9 genomes available as of August 1, 2018, from the National Center for Biotechnology Information (NCBI) Reference Sequence Database (https://www.ncbi.nlm.nih.gov/RefSeq), which included information on source, geographic location, and collection year.

### WGS and Bioinformatic Analyses

We prepared DNA for multiplexed, paired-end sequencing by preparing libraries using either the Nextera XT DNA Library Preparation Kit (Illumina, Inc.), according to manufacturer instructions, or the Kapa Hyper Prep Kit (Kapa Biosystems, Inc., https://www.sigmaaldrich.com) and uniquely barcoded adaptors from NEXTFLEX-96 Unique Dual Index barcodes (Bioo Scientific, https://www.biooscientific.com). We prepared equimolar pools of *S. aureus* libraries at a concentration of 2 nmol and sequenced on a MiSeq (Illumina, Inc., https://ww.illumina.com) at 2 × 300 bp. WGS data are available in the NCBI Sequence Read Archive (http://www.ncbi.nlm.nih.gov; BioProject no. PRJNA574434).

We used SPAdes (*13*) to generate de novo assemblies and compared these against the *S*. *aureus* MLST database (*14*) to assign MLSTs. We used ABRicate (https://github.com/tseemann/abricate) to search the ResFinder database for antimicrobial-resistance (AMR) genes (*15*). We used BLASTN (https://blast.ncbi.nlm.nih.gov/Blast.cgi) to detect genes in the phage-associated immune evasion cluster (IEC), including *scn*, *chp*, *sak*, *sea* (GenBank accession no. NC_009641), and *sep* (GenBank accession no. BA000018) (*16*).

We used the NASP pipeline (*17*) to map sequence reads against the de novo-assembled genome of North Carolina isolate IHOW6.1 (BioProject accession no. PRJNA574434) and to perform single-nucleotide polymorphism (SNP) calling, as described previously (*8*). We used Gubbins version 2.3.1 (*18*) to remove recombination from the SNP alignment and used the remaining SNPs in the core genome to construct a midpoint-rooted maximum-likelihood tree by using PhyML (*19*) with a general time-reversible model of nucleotide substitution and 100 bootstrap replicates (*20*). We used the same methods to perform a separate SNP analysis of the cluster containing the North Carolina isolates (clade 3) to improve the resolution of the transmission analysis. We calculated pairwise SNP differences by using MEGA5 (*21*). To define a SNP-based threshold for assigning isolates into putative transmission clusters, we used the maximum within-farm pairwise SNP distance among *S. aureus* CC9 isolates from IHO-1, in which all isolates were collected from the same IHO at the same sampling time.

### Antimicrobial Susceptibility Testing

Isolates in the North Carolina collection previously were assessed for susceptibility to a panel of antimicrobial drugs by using the Phoenix Automated Microbiology System (Becton Dickinson, https://www.bd.com) or the Kirby-Bauer disk diffusion method (Appendix Table 2). Testing was completed by the Clinical Microbiology Laboratory at the Johns Hopkins Hospital based on Clinical Laboratory Standards Institute (CLSI; https://clsi.org) guidelines specified in the source studies (*1*–*3*) (Appendix Table 2). We defined MDRSA as *S. aureus* isolates resistant to >3 classes of antimicrobial drugs (*22*). We defined MRSA as *S. aureus* harboring the *mecA* gene.

### Statistical analysis

We used the χ^2^ test to compare AMR and IEC genes between groups. We performed all statistical analyses by using Stata version 14.2 (StataCorp LLC, https://www.stata.com).

## Results

**Figure 1:**
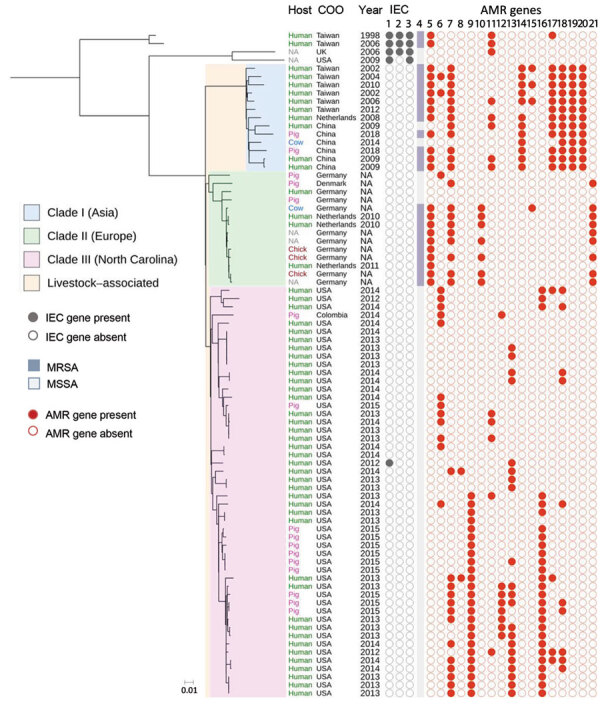
Maximum-likelihood tree demonstrating population structure of *Staphylococcus aureus* clonal complex (CC) 9 isolates from humans and livestock in North Carolina, USA, and reference sequences. A total of 81 *S. aureus* CC9 isolates from human and livestock specimens were included in this midpoint-rooted maximum-likelihood phylogeny based on 3,847 core genome single-nucleotide polymorphisms. *S. aureus* isolates belonged to 3 phylogeographically distinct clades (C1–C3). All the North Carolina collection isolates were included in C3. IEC genes are shown in columns 1, *scn*; 2, *sak*; and 3, *chp.* MRSA is shown in column 4. AMR genes are shown in columns 5, *mecA*; 6, *tet*(K); 7, *tet*(L); 8, *tet*(T); 9, *erm*(A); 10, *erm*(B); 11, *erm*(C); 12, *vga*(A)_LC_; 13, *lnu*(A); 14, *lnu*(B); 15, *str*; 16, *spc*; 17, *aadD*; 18, *aac*(6); 19, *ant*(6)-1a; 20, *dfr*G; and 21, *dfr*K. Scale bar indicates nucleotide substitutions per site. AMR, antimicrobial resistance; Chick, chicken; COO, country of origin; IEC, immune evasion cluster; MRSA, methicillin-resistant *S. aureus*; MSSA, methicillin-susceptible *S. aureus*; NA, not applicable.

All 49 isolates from the North Carolina collection used in WGS analysis were classified as sequence type 9 by MLST. Among 81 *S. aureus* CC9 isolates analyzed, 95% (77/81) were located in 3 major clades, C1, C2, and C3 (Figure 1; Appendix Tables 3, 4). Despite the small number of pig isolates, each clade contained both pig and human isolates (Figure 1; Appendix Table 3).

Among C1–C3 isolates, 61% (47/77) contained tetracycline resistance genes. By contrast, only 1 (1.3%) of the isolates in C1–C3 contained IEC genes (Figure 1; Appendix Table 4). The presence of pig isolates coupled with the absence of IEC genes and presence of tetracycline resistance genes in C1–C3 suggest that C1–C3 isolates may be members of a larger LA-SA CC9 clade. LA-MRSA CC9, which harbored the *mecA* gene, was present in C1 and C2 but absent from C3.

C1 was composed of isolates primarily originating from Asia (12/13 isolates; 92%), of which 46% (6/13) were from China and 46% (6/13) were from Taiwan (Figure 1; Appendix Table 3). All of C2 (14/14 isolates) was composed of isolates originating from Europe, of which 71% (10/14) were from Germany, 21% (3/14) were from the Netherlands, and 7% (1/14) were from Denmark (Figure 1; Appendix Table 3). C3 included 100% (49/49) of the North Carolina isolates, which made up 98% (49/50) of all C3 isolates (Figure 1; Appendix Table 3). Only 2 isolates grouped into a clade that did not correspond to the continent of predominance within the clade. A single isolate from Colombia (South America) grouped into C3 with isolates from North Carolina, and a single isolate from the Netherlands grouped into C1 with isolates from Asia (Figure 1; Appendix Table 3).

**Figure 2:**
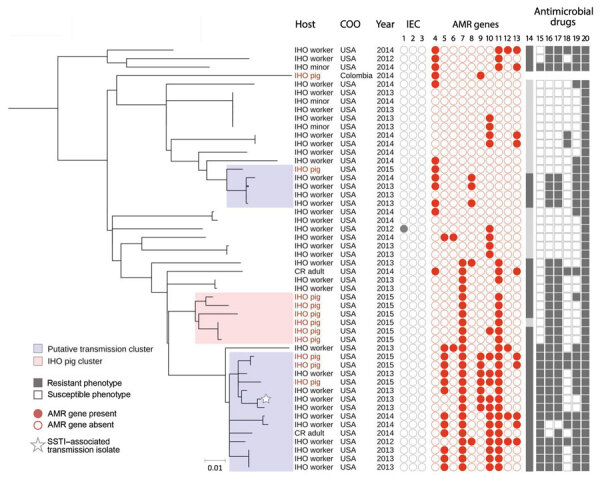
High-resolution population structure of clade 3 livestock-associated *Staphylococcus aureus* clonal complex (CC) 9 isolates from humans and livestock in North Carolina, USA, and reference isolates. A subset of 50 livestock-associated *S. aureus* CC9 isolates that were collected from IHO pigs, IHO workers, IHO minors, and CR adults were included in this midpoint-rooted maximum-likelihood phylogeny based on 1,198 core genome single-nucleotide polymorphisms. A single subclade, denoted as the IHO pig cluster, included only pig isolates from IHO-1 and was used to set a threshold of 43 SNPs for identifying transmission clusters; clusters of IHO pig and human isolates separated by <43 SNPs are considered transmission clusters. Two subclades included intermingled human and IHO pig isolates with a high degree of phylogenetic relatedness and were considered transmission clusters. IEC isolates are shown in columns 1, *scn*, 2, *sak*, and 3, *chp*. AMR genes are shown in columns 4, *tet*(K); 5, *tet*(L); 6, *tet*(T); 7, *erm*(A); 8, *erm*(C); 9, *vga*(A)_LC_; 10, *lnu*(A); 11, *spc*; 12, *aadD*; and 13, *aac*(6). MDRSA is shown in column 14. Antimicrobial drug resistance is shown in columns 15, fluoroquinolone resistance, considered phenotypic resistance to moxifloxacin; 16, lincosamide resistance, considered phenotypic resistance to clindamycin; 17, macrolide resistance, considered phenotypic resistance to erythromycin; 18, aminoglycoside resistance, considered phenotypic resistance to gentamicin; 19, tetracycline resistance, considered phenotypic resistance to tetracycline; and 20, penicillin resistance. Scale bar indicates nucleotide substitutions per site. AMR, antimicrobial resistance; CR, community resident, a person with no known exposure to livestock; IHO, industrial hog operation; MDRSA, multidrug resistant *S. aureus;* SSTI, skin and soft tissue infection.

High-resolution phylogenetic analysis of C3 revealed multiple distinct subclades, one of which contained all 6 IHO-1 pig isolates from North Carolina (Figure 2). The pairwise SNP distance among IHO pig isolates from IHO-1 ranged from 0–43 SNPs. Thus, we used 43 SNPs as the threshold to identify putative transmission clusters and found that 19 isolates fell into 2 distinct putative transmission clusters (Figure 2). The minimum pairwise SNP distance between IHO pig and human isolates within putative transmission clusters ranged from 12–34 SNPs.

Almost all (94.7%) transmission cluster isolates were classified as MDRSA (Figure 2). Among 19 putative transmission cluster isolates, 14 were recovered from IHO workers, all of which were classified as MDRSA. Among IHO worker isolates, 2 differed from an IHO pig isolate by only 12 SNPs. An IHO worker isolate that was associated with a recent SSTI differed from an IHO pig isolate by only 20 SNPs (Figure 2). One transmission cluster isolate was from an adult community resident with no known exposure to livestock; this isolate also was classified as MDRSA (Figure 2). The minimum SNP distance between this isolate and the closest IHO pig isolate was 25 SNPs and it was 22 SNPs from the closest IHO worker isolate. Among 3 isolates from minors, 2 were identical (0 SNP differences) to an isolate from an IHO worker in the same household (Figure 2); 1 of the isolates from a minor was collected at the same sampling time as the IHO worker isolate. Among C3 isolates, we noted genetic determinants conferring resistance to tetracyclines, including *tet*(K), *tet*(L), *tet*(T); macrolides, including *erm*(A), *erm*(C); lincosamides, including *lnu*(A); aminoglycosides, *aac6’-aph2”*, *spc*, and *aadD*; and streptogramins, including *vga*(A)_LC_ (Figure 2).

We noted abundant genetic determinants conferring resistance to several antimicrobial classes among C3 isolates, including tetracyclines in 50% (25/50), macrolides in 56% (28/50), and aminoglycosides in 62% (31/50) of C3 isolates (Figure 2; Appendix Table 4). Among LA-SA CC9 clades, 50% (25/50) of C3 isolates were uniquely enriched for *erm*(A) genes, 16% (8/50) for *vga*(A)_LC_, 42% (21/50) for *lnu*(A), and 54% (27/50) for *spc* (Figure 1; Appendix Table 4). The *mecA* gene was absent from C3 but common among C1 and C2 isolates.

## Discussion

Our WGS analysis suggests that the clonal expansion of LA-SA CC9 in North Carolina is distinct from that in Asia and Europe and that LA-SA CC9 from IHO pigs and humans in high-density pig-producing counties of North Carolina come from a common pool. Considering the high degree of phylogenetic relatedness among intermingled IHO pig and human isolates in putative transmission clusters, the results of this study support potential transmission of antimicrobial-resistant LA-SA CC9 between IHO pigs and humans in the United States.

Our results also provide evidence of household-level transmission of LA-SA CC9 between IHO workers and minors and suggest that potential LA-SA CC9 transmission is not limited to the occupational setting. Dissemination of LA-SA CC9 into the general human population represents a public health concern for 2 reasons. Globally, communities include a higher proportion of children, the elderly, and probably immunocompromised persons, who are at higher risk of developing invasive staphylococcal infections, compared with IHO workers who are predominantly healthy adults of working age. Our analysis revealed an 11-year-old child and an IHO worker residing in the same household who were carrying identical LA-SA CC9 isolates (0 SNP differences) at the same sampling time, which provides strong evidence of household transmission of LA-SA CC9 between IHO workers and their children. Second, clinical implications might arise regarding treatment regimens for LA-MDRSA CC9 colonization and infection. Most (63.3%; 31/49) LA-SA CC9 isolates from North Carolina were multidrug-resistant and carried multiple genes conferring resistance to antimicrobial drug classes critical for human medicine (*23*). Of note, the single LA-SA CC9 isolate from an IHO worker who reported a recent SSTI belonged to a putative transmission cluster, displayed an MDRSA phenotype, and previously was reported to display a high degree of pathogenicity compared with a hypervirulent community-associated MRSA strain, USA300 (GenBank accession no. CP000255), in a mouse model of SSTI (*24*).

Our results support potential transmission of LA-SA CC9 between IHO pigs and humans, and between humans and other humans, in the top 10 pig-producing counties in North Carolina. These findings are consistent with previous publications on LA-SA CC9 and other lineages of LA-SA*.* First, a separate analysis of LA-MRSA CC9 recovered from IHO pigs in China suggested potential transmission of LA-MRSA CC9 between pigs, humans, and cows (*11*). Second, an abundance of previous epidemiologic and WGS analyses support transmission of diverse lineages of LA-SA from pigs to humans, which can result in human SSTI and bloodstream infections (*8*,*10*,*25*). Last, prior WGS analyses and epidemiologic studies have provided support for household transmission of LA-SA CC9 and CC398 between persons based on spatial, temporal, and genotypic overlap (*2*,*3*,*26*). In our analysis, the exact transmission pathway remains unclear because we did not ascertain the direction of transmission or whether transmission occurred through direct or indirect contact.

Previous studies have suggested a *S. aureus* mutation rate of 5–10 SNPs per year per genome (*27*–*30*), but our threshold of 43 SNPs was justified for 2 reasons. First, our empirically derived SNP threshold was consistent with SNP-based thresholds used by others to identify suspected transmission of MRSA in clinical settings (*31*) and previous measures of within-person *S. aureus* diversity (*32*). The robustness of our findings was supported when we used the median (32 SNPs), rather than maximum (43 SNPs), pairwise SNP distance among IHO pig cluster isolates as the SNP threshold for identification of putative transmission clusters. We excluded only 1 isolate from an IHO worker from putative transmission clusters, and the excluded isolate was not the SSTI-associated isolate (data not shown). Second, the aim of this study was to clarify whether any SNP-based evidence of transmission between IHO pig and human populations in North Carolina exists, rather than provide evidence of recent or incident transmission or to identify specific pathways of transmission. Using 43 SNPs as the threshold enabled us to observe potential direct or indirect transmission that might not be observed by using epidemiologic data alone. Investigations of *S. aureus* transmission conventionally combine epidemiologic and strain typing data, but these methods can fail to identify transmission links in cases in which spatial and temporal overlap is lacking (*31*). Using the epidemiologic data that were available to us, such as multiple *S. aureus* CC9 isolates from the same IHO, household, or individual, we observed SNP-based evidence of *S. aureus* CC9 clustering that would be expected biologically (Appendix Table 5).

Since 2016, tetracyclines have been the most heavily used antimicrobial drug class in the US pig production system, followed distantly by macrolides, lincosamides, aminoglycosides, streptogramins, and fluoroquinolones (*33*,*34*). If antimicrobial-resistant CC9 strains were enriched through selective pressure, antimicrobial use in pig production possibly has played a role in the clonal expansion of LA-SA CC9 in North Carolina and other regions of the world. Of note, resistance to several of these antimicrobial drug classes was conferred by different AMR genes in C1, C2, and C3 (Figure 1; Appendix Table 4), highlighting different evolutionary pathways for adaptation to antimicrobial selection pressures in different regions of the world. Continued surveillance of IHO pigs and humans, including during and after regulatory and policy restrictions on antimicrobial use in animal agriculture, could provide critical insight into the potential contribution of antimicrobial use in the clonal expansion of LA-SA CC9 and its associated AMR genes in the United States.

The strengths of our study included using SNP-based analyses to examine the population structure and transmission dynamics of LA-SA CC9 among pigs and humans in a region of North Carolina with the highest density of IHOs in the United States (*35*), a region in which residents and IHO workers are actively expressing concerns about IHO-related exposures (*36*). Second, our study used SNP distance to classify human isolates closely related to IHO pig isolates, which is an improvement on previous studies that used *spa-*typing, MLST typing, absence of IEC genes (specifically *scn*), phenotypic AMR determination, or combinations of these techniques, to classify *S. aureus* isolates as livestock-associated (*2*,*3*,*12*). Third, the use of a SNP-based definition for cluster analysis can capture the potential for transmission between animal and human populations that would have been missed by using more conventional epidemiologic methods alone (*31*).

Limitations of our study included that we were not able to provide evidence for directionality of transmission. We rooted our high-resolution phylogenetic tree at the midpoint; therefore, we are unsure if the most ancestral clade of *S. aureus* CC9 is of human or animal origin. In addition, whereas the SNP-based evidence for pig-to-human transmission could have been strengthened by spatial or temporal data linking pigs and workers at the same IHO, these data were not available because of efforts to protect the privacy of participants enrolled in the epidemiologic studies and because of limited access to US IHOs in the Unites States (*37*). In contrast to countries in Europe, the lack of access to IHOs prevents us from assessing the generalizability of our results in the United States. We hypothesize that we would see even closer genetic relatedness between IHO worker and IHO pig LA-SA CC9 isolates collected from the same IHO at the same time. Last, our collection of *S. aureus* CC9 isolates was limited. The North Carolina collection was a convenience sample that identified *S. aureus* CC9 isolates from only 6 IHOs, which does not represent the full population of IHOs or pigs in North Carolina. Also, we excluded many isolates selected for WGS from SNP-analysis because they did not pass our quality control criteria (Appendix), potentially introducing bias into the studied isolate sample. Additional *S. aureus* CC9 isolates likely are available now in the NCBI Reference Sequence Database, but publicly available LA-SA CC9 sequence data were limited when we accessed the database for this study. A more representative dataset could provide more refined estimates on frequency of transmission in North Carolina and other regions of the world.

Despite these limitations, our results show a high degree of phylogenetic relatedness between IHO pig and human LA-SA CC9 isolates in the top 10 pig-producing counties in North Carolina. The presence of a highly pathogenic SSTI-associated LA-SA CC9 isolate with an MDRSA phenotype in a putative transmission cluster warrants future investigations into the disease burden associated with these strains in the United States. Future research could further improve or build on our findings by including environmental isolates and considering WGS analysis in conjunction with spatial and temporal data analysis to investigate the frequency of transmission, environmental exposure routes, and geographic extent of LA-SA CC9. Our reference dataset might be useful in future investigations of worker and community health concerns related to LA-SA CC9 dissemination and acquisition, both in North Carolina and in other regions of the United States with high densities of IHOs.

Appendix Additional information on methods for collecting and sequencing *Staphylococcus aureus* clonal complex 9 isolates from humans and pigs raised in industrial hog operations, North Carolina, United States.
