# Supplementary material for: Transmission of Antimicrobial-Resistant Staphylococcus aureus Clonal Complex 9 between Pigs and Humans, United States
Source: Emerg Infect Dis. 2021 Mar;27(3):740–8. doi: 10.3201/eid2703.191775 (PMC7920674; doi:10.3201/eid2703.191775)
Supplement: Appendix — Additional information on methods for collecting and sequencing Staphylococcus aureus clonal complex 9 isolates from humans and pigs raised in industrial hog operations, North Carolina, United States. [file 19-1775-Techapp-s1.pdf]

Article DOI: https://doi.org/10.3201/eid2703.191775

Transmission of Antimicrobial-Resistant *Staphylococcus aureus* CC9 between Pigs and Humans, United States

Appendix

Sources of *Staphylococcus aureus* clonal complex 9 Isolates from Humans and from Pigs Raised in Industrial Hog Operations, North Carolina

We collected *Staphylococcus aureus* clonal complex 9 (CC9) isolates from industrial hog operation (IHO) pigs from a convenience sample of a single IHO in North Carolina (IHO-1), as described previously (*1*), and from hanging a length of undyed, unbleached cotton ropes in pig pens at IHO-2–IHO-21 (Appendix Table 1). Isolates collected from IHO2–IHO-21 have not been published previously. For IHO-1 pig isolates, we used Copan E-swabs for sample collection. Each pig was swabbed in the right nares, right side of mouth, skin behind the right ear, right perineal mucosa, and any observed skin lesion. The swabs were subjected to broth enrichment culture, as previously described (*1*).

For IHO-2–IHO-21, we collected samples of oral fluids from pigs in pens by hanging ropes. The ropes were hung in pens away from feed or water and the pigs could chew on the rope, which collected oral fluids in the cotton material. We extracted oral fluids from the ropes by squeezing the rope contents into a centrifuge tube, centrifuging at 500 RCF for 10 min, and transferring the supernatant (eluate) into a clean tube. IHO pig oral secretion eluates were processed for *S. aureus* following the procedures for detection of *S. aureus* from human nasal swabs (*2*,*3*). The *spa* type for all IHO pig isolates was characterized, as previously described (*1*), and used to assign each isolate to a putative multilocus sequence type (MLST).

For human isolates, we used *S. aureus* isolates collected from participants who were previously enrolled into 1 of 3 separate epidemiologic studies (study 1, study 2, and study 3) and screened for nasal carriage of *S. aureus* (Appendix Table 1) (*2*–*4*). Sample collection, sample

processing, and *S. aureus* isolation methods were described previously (*2*–*4*). Study 1 was cross-sectional and surveyed 99 industrial livestock operation workers and their household contacts and 105 workers from antibiotic-free livestock operations and their household contacts for *S. aureus* nasal carriage at a single time point (*4*). Study 2 was longitudinal and followed 183 IHO workers and their adult and minor (7–17 years of age) household contacts for 4 months and assessed *S. aureus* nasal carriage at baseline and 8 biweekly (once every 2 weeks) time points (*3*). Study 3 was cross-sectional and assessed *S. aureus* nasal carriage among 800 participants living in one of the top 10 pig-producing counties of North Carolina, including 198 households with 1 IHO worker and 1 IHO minor <7 years of age and 202 households with 1 community resident adult and 1 community resident minor <7 years of age who had no known exposure to livestock (*2*). MLST was previously determined for all study 1 isolates (*4*). The *spa* type was previously characterized for study 2 and study 3 isolates and used to assign a putative MLST based on previously published associations between *spa* type and MLST (*2*,*3*).

Selection of *S. aureus* CC9 Isolates for Whole-Genome Sequencing Analysis

We attempted to select a representative subset of CC9 isolates recovered from 21 IHOs in North Carolina. From IHO-1, we selected ≥1 isolate per pig life stage per barn, resulting in 11 putative CC9 isolates selected for whole-genome sequencing (WGS). For IHO-2–IHO-21, *S. aureus* was recovered from 5 hanging rope samples from 5 different IHOs (IHO-2, IHO-3, IHO-4, IHO-5, and IHO-6). *S. aureus* CC9 isolates representing each identified CC9-related *spa* type (1 isolate per *spa* type) from a given IHO were selected, resulting in 5 putative *S. aureus* CC9 isolates from 4/5 *S. aureus* positive IHOs being subjected to WGS.

In study 1, 3 putative *S. aureus* CC9 isolates were recovered from 3 IHO workers, which were all selected for WGS. In study 2, 126 putative *S. aureus* CC9 isolates were recovered, including 123 from 38 IHO-workers and 1 each from 3 IHO minors. To evaluate persistent colonization and potential evidence for household transmission of CC9, we focused our selection of putative CC9 isolates on IHO workers carrying putative CC9 isolates at a minimum of 2 time points; IHO workers and their household contacts carrying putative CC9 isolates at the same time point; and household contacts of IHO workers. This resulted in 63 CC9 isolates being selected for WGS from study 2. In study 3, 16 putative *S. aureus* CC9 isolates were recovered

from humans. Because we were interested in evaluating evidence for potential transmission of CC9 between pigs, IHO workers, and community residents living near IHOs, we focused our selection of isolates on putative CC9 isolates from community residents and IHO workers residing in those communities. Three putative CC9 isolates were identified in 2 community residents and 1 IHO worker, which were all selected for WGS. In total, this isolate selection framework resulted in a collection of 16 putative CC9 isolates from IHO pigs and 69 putative CC9 isolates from humans.

After performing WGS, we applied a stringent quality control threshold defined as <5% unclassified reads (minimal contamination) and $>2.0 \times 10^6$ bp at 25× coverage, which removed 6 IHO pig isolates and 30 human isolates from subsequent analysis. We were not able to resequence these isolates because of financial constraints, resulting in 49 isolates in the final North Carolina collection. The final North Carolina collection used in SNP analysis consisted of 10 isolates from pigs raised on 4 different IHOs, 34 isolates from 25 IHO workers, 3 isolates from 3 IHO minors, and 2 isolates from 2 adult community residents (Appendix Table 1).

References

1. Davis MF, Pisanic N, Rhodes SM, Brown A, Keller H, Nadimpalli M, et al. Occurrence of *Staphylococcus aureus* in swine and swine workplace environments on industrial and antibiotic-free hog operations in North Carolina, USA: a One Health pilot study. Environ Res. 2018;163:88–96. PubMed https://doi.org/10.1016/j.envres.2017.12.010

2. Hatcher SM, Rhodes SM, Stewart JR, Silbergeld E, Pisanic N, Larsen J, et al. The prevalence of antibiotic-resistant *Staphylococcus aureus* nasal carriage among industrial hog operation workers, community residents, and children living in their households: North Carolina, USA. Environ Health Perspect. 2017;125:560–9. PubMed https://doi.org/10.1289/EHP35

3. Nadimpalli ML, Stewart JR, Pierce E, Pisanic N, Love DC, Hall D, et al. Face mask use and persistence of livestock-associated *Staphylococcus aureus* nasal carriage among industrial hog operation workers and household contacts, USA. Environ Health Perspect. 2018;126:127005. PubMed https://doi.org/10.1289/EHP3453

4. Rinsky JL, Nadimpalli M, Wing S, Hall D, Baron D, Price LB, et al. Livestock-associated methicillin and multidrug resistant Staphylococcus aureus is present among industrial, not antibiotic-free livestock operation workers in North Carolina. PLoS One. 2013;8:e67641. PubMed https://doi.org/10.1371/journal.pone.0067641

Appendix Table 1. Selection of putative and MLST-confirmed *Staphylococcus aureus* CC9 isolates from North Carolina, United States*

	Source									
	IHO pig isolates, no. (%)						Human isolates, no. (%)			
Group	IHO-1	IHO-2	IHO-3	IHO-4	IHO-5	IHO-6	Study 1	Study 2	Study 3	Total, no. (%)
IHO pig	6/37 (16)	0/9 (0)	0/13 (0)	1/10 (10)	2/10 (20)	1/12 (8)	–	–	–	10/91 (11)
IHO worker	–	–	–	–	–	–	3/3 (100)	30/123 (24)	1/12 (8)	34/138 (25)
IHO minor	–	–	–	–	–	–	–	3/3 (100)	0/1 (0)	3/4 (75)
CR adult	–	–	–	–	–	–	–	–	2/2 (100)	2/2 (100)
CR minor	–	–	–	–	–	–	–	–	0/1 (0)	0/1 (0)

* MLST, multilocus sequence type; CC9, clonal complex 9; IHO, industrial hog operation; CR, community resident; –, no isolates.

Appendix Table 2. Antimicrobial drug susceptibility testing methods and guidelines used for interpreting phenotypic antimicrobial resistance*

Source	Method	CLSI guideline, y
Study 1	Kirby-Bauer disk diffusion	2010
Study 2	Kirby-Bauer disk diffusion	2013
Study 3	Phoenix Automated Microbiology System†	2015
IHO-1	Kirby-Bauer disk diffusion	2012, 2014
IHO-2 to IHO-21	Kirby-Bauer disk diffusion	2015

*CLSI, Clinical and Laboratory Standards Institute; IHO, industrial hog operation.
†Becton Dickinson Diagnostic Systems, https://www.bd.com.

Appendix Table 3. Epidemiologic characteristics of *Staphylococcus aureus* CC9 isolates*

	Clade, no. (%)					
Characteristic	C1	C2	C3	C1–C3	Other, no. (%)	Total, no. (%)
Source						
Human	10 (77)	4 (29)	39 (78)	53 (69)	2 (50)	55 (68)
Pig	2 (15)	3 (21)	11 (22)	16 (21)	0	16 (20)
Chicken	0	3 (21)	0	3 (3.9)	0	3 (4)
Cow	1 (8)	1 (7)	0	2 (2.6)	0	2 (2)
Unknown	0	3 (21)	0	3 (3.9)	2 (50)	5 (6)
Country						
United States	0	0	49 (98)	49 (64)	1 (25)	50 (62)
Colombia	0	0	1 (2)	1 (1)	0	1 (1)
Germany	0	10 (71)	0	10 (13)	0	10 (12)
Netherlands	1 (8)	3 (21)	0	4 (5)	0	4 (5)
Denmark	0	1 (7)	0	1 (1)	0	1 (1)
China	6 (46)	0	0	6 (8)	0	6 (7)
Taiwan	6 (46)	0	0	6 (8)	2 (50)	8 (10)
United Kingdom	0	0	0	0	1 (25)	1 (1)
Collection						
North Carolina	0	0	49 (98)	49 (64)	0	49 (60)
Reference	13 (100)	14 (100)	1 (2)	28 (36)	4 (100)	32 (40)

*CC9, clonal complex 9.

Technical Appendix Table 4. Molecular characteristics of *Staphylococcus aureus* CC9 isolates*

Gene	Clade, no. (%)							C1–C3 vs. other, p value†	C3 vs. C1–C2, p value†
	C1, n = 13	C2, n = 14	C3, n = 50	C1–3, n = 77	C1–2, n = 27	Other, n = 4	Total, no. (%), n = 81		
Immune evasion cluster									
scn	0	0	1 (2)	1 (1)	0	4 (100)	5 (6)	0.0001	–
sak	0	0	0	0	0	3 (75)	3 (4)	0.0001	–
chp	0	0	0	0	0	4 (100)	4 (5)	0.0001	–
Methicillin resistance									
mecA	11 (85)	10 (71)	0	21 (27)	21 (78)	2 (50)	23 (28)	–	0.0001
Tetracycline resistance									
tet(K)	2 (15)	1 (7.1)	12 (24)	15 (19)	3 (11)	0	15 (19)	–	–
tet(L)	12 (92)	9 (64)	13 (26)	34 (44)	21 (78)	0	34 (42)	–	0.0001
tet(T)	0	0	2 (4)	2 (3)	0	0	2 (3)	–	–
≥1 gene	12 (92)	10 (71)	25 (50)	47 (61)	22 (81)	0	47 (58)	0.028	0.008
Macrolide resistance									
erm(A)	0	0	25 (50)	25 (32)	0	0	25 (31)		0.0001
erm(B)	0	7 (50)	0	7 (9)	7 (26)	0	7 (9)	–	0.0001
erm(C)	5 (38)	0	5 (10)	10 (13)	5 (19)	3 (75)	13 (16)	0.012	–
≥1 gene	5 (38)	7 (50)	28 (56)	40 (52)	12 (44)	3 (75)	43 (53)	–	–
Streptogramin B resistance									
vga(A)$_{LC}$‡	0	0	8 (16)	8 (10)	0	0	8 (10)	–	0.045
Lincosamide resistance									
lnu(A)	0	0	21 (42)	21 (27)	0	0	21 (26)	–	0.0001
lnu(B)	12 (92)	0	0	12 (16)	12 (44)	0	12 (15)	–	0.0001
≥1 gene	12 (92)	0	21 (42)	33 (43)	12 (44)	0	33 (41)	–	–
Aminoglycoside resistance									
str	3 (23)	1 (7)	0	4 (5)	4 (15)	0	4 (5)	–	0.013
ant(6)-la	13 (100)	0	0	13 (17)	13 (48)	0	13 (16)	–	0.0001
aac(6")-aph(2")	13 (100)	0	10 (20)	23 (30)	13 (48)	0	23 (28)	–	0.018
spc	0	0	27 (54)	27 (35)	0	0	27 (33)	–	0.0001
aadD	12 (92)	0	4 (8)	16 (21)	12 (44)	1 (25)	17 (21)	–	0.0001
≥1 gene	13 (100)	1 (7)	31 (62)	45 (58)	43 (56)	1 (25)	46 (57)	–	–
Trimethoprim resistance									
dfrG	13 (100)	0	0	13 (17)	13 (48)	0	13 (16)	–	0.0001
dfrK	0	9 (64)	0	9 (12)	9 (33)	0	9 (11)	–	0.0001
≥1 gene	13 (100)	9 (64)	0	22 (29)	22 (81)	0	22 (27)	–	0.0001

*CC9, clonal complex 9.
†Only $p<0.05$ are shown; –, $p\geq0.05$
‡Also encodes lincosamide and pleuromutilin resistance.

Appendix Table 5. Maximum pairwise SNP distances in *Staphylococcus aureus* between within-farm, within-household, and within-person isolates*

Setting	No. isolates	Maximum pairwise SNP distance, no.
Within-farm		
IHO-1	6	43
Within-household		
Household A	2	1
Household B	2	4
Household C	2	93
Household D	3	0
Within-person		
Participant A	2	6
Participant B	2	0
Participant C	2	1
Participant D	4	21
Participant E	2	0
Participant F	3	2

*SNP, single-nucleotide polymorphism; IHO, industrial hog operation.
